# Ranking’s Half-Yearly Abstract of the Medical Sciences

**Published:** 1853-02

**Authors:** 


*Ranking's Half-Yearly Abstract of the Medical Sciences.*—This very complete abstract of the improvements and discoveries in practical medicine, continues to appear with its accustomed regularity. The American reprint, by Lindsay & Blakiston, is furnished to mail subscribers, free of postage, at $2,00 per annum.

